# Preliminary Adaptation of Motor Tests to Evaluate Fine Motor Skills Associated with Mathematical Skills in Preschoolers

**DOI:** 10.3390/ejihpe13070098

**Published:** 2023-07-20

**Authors:** Pedro Flores, Eduarda Coelho, Maria Isabel Mourão-Carvalhal, Pedro M. Forte

**Affiliations:** 1 CI-ISCE, ISCE Douro, 4560-708 Penafiel, Portugal; pedro.miguelforte@iscedouro.pt; 2 Sports Department, University of Trás-os-Montes and Alto Douro, 5000-801 Vila Real, Portugal; 3 Research Center in Sports, Health and Human Development, 6201-001 Covilhã, Portugal; 4 Department of Sports, Instituto Politécnico de Bragança, 5300-253 Bragança, Portugal

**Keywords:** preschool, motor tests, fine motor skills, mathematical skills, identification, selection, adaptation, validation

## Abstract

Mathematics is the subject in which many school-age children reveal difficulties. The literature has shown that fine motor skills, namely fine motor coordination and visuomotor integration, have been more robustly associated with mathematical performance. Studies have shown the importance that instruments have to evaluate these skills, however, the characteristics of these instruments do not fit the reality of kindergartens, they are usually time consuming and expensive and are usually administered by specialists. Thus, the main objective of this study was to identify, select, adapt and validate motor tests to evaluate fine motor skills associated with mathematical skills to allow the kindergarten teachers to apply them simultaneously to the class, with few material resources, in a short period of time and without the need for a lot of training to apply, score and classify. For this purpose, firstly, it was necessary to understand the main difficulties highlighted by kindergarten teachers regarding the use of instruments to evaluate fine motor skills and, thus, elaborate criteria to identify and select the tests that best fit the reality of kindergartens. The test identified, selected and adapted to evaluate fine motor coordination was threading beads from the Movement Evaluation Battery for Children, 2nd Edition. The main adaptation of the test was related to time, that is, instead of counting the time it takes the child to string the total number of cubes on the string, we counted the number of cubes the child strung on the string in a pre-defined time. To evaluate visual–motor integration, the test identified, selected and adapted was the Visual–Motor Integration (6th Edition) test. The main adaptation was related to material resources, that is, it will be possible to apply the test using only one sheet per child instead of the seven suggested by the original test. After the preliminary adaptation of the tests, their validation was performed by means of the degree of reliability (test-retest) and predictive validity. The results indicated that the adapted tests presented an excellent degree of reliability (>0.9) and could therefore be used to administer them simultaneously to the class group. The adapted Visual–Motor Integration test seems to be the most suitable one to be used by kindergarten teachers, in a classroom context, to simultaneously evaluate students’ fine motor skills and associate their results with mathematical skills.

## 1. Introduction

There is evidence suggesting an interrelationship between motor and cognitive development [1,2]. Several investigations have highlighted the importance of movement in cognition, namely in the performance of mathematical skills such as abstract cognitive representations in general and in improving basic numerical representations in particular [3]. As Fischer et al. [4] propose, numbers are embodied concepts and not abstractions dissociated from sensory experiences. Furthermore, the theory of embodied cognition assumes that certain cognitive and motor areas of the brain are activated simultaneously when solving mathematical problems [5].

Mathematics plays a major role in the school curriculum, is fundamental to learning [6,7] and can be an indicator of future academic and professional success [8]. However, it is the subject in which many school-age children experience difficulties [9,10,11,12]. Fundamental learning for the development of mathematical skills in preschool education consists of two main areas, numbers and geometry and measurement [13].

Recent systematic review studies concluded that fine motor skills (FMS) justified mathematical academic performance in preschool children [14,15,16].

These skills usually coincide with fine motor coordination (FMC), which refers to movements involving eye–hand coordination (eye–hand), manual dexterity, motor sequencing and speed and accuracy. This is exhibited in tasks such as tracing, finger tapping, building with legos/blocks, moving coins from one place to another or inserting them into a slot and stringing beads. These skills are often present among other skills that refer to the precision of movement [17,18], which may also be referred to as non-graphomotor skills [19], and along with motor and visual integration or visuomotor integration (VMI) and/or visuospatial integration, which refers to the organization of small muscle movements of the hand and fingers by processing visual and spatial stimuli, relying more on synchronized hand–eye movements [17,18]. These skills are typically exhibited in tasks of writing, drawing and copying shapes, letters or other stimuli [20] and may be referred to as graphomotor skills [19].

Although the literature has shown that VMI is part of the FMS, a skill standing out in association with mathematical skills [14] does not mean that this skill will be the one that can most predict math performance, since studies that included both FMS concluded that both FMC and VMI were predictors of math performance [21,22,23,24]. Studies using only FMC concluded that this motor skill was associated with mathematics performance [4,25,26] or VMI [27,28,29,30,31,32,33]. Thus, each FMS was used depending on the purpose of each study [14].

In one systematic review, one of the objectives of which was to identify the instruments used to evaluate FMS associated with mathematical skills in typically developing children attending preschool education, they concluded that the main characteristics of the instruments used in the studies showed that copying picture or drawing tasks were the most commonly used to evaluate VMI and object-handling tasks with pincer-like movements were the most commonly used to evaluate FMC [14]. However, despite the fact that the literature highlights the importance that the instruments used to evaluate FMS have been screened for possible difficulties associated with mathematics performance, most of these studies used direct neuropsychological evaluations, recognizing that these instruments are usually time-consuming and expensive and are usually only administered by specialists for a certain purpose [34,35]. In this sense, the characteristics of the most commonly used instruments that evaluate FMS for diagnosing mathematics performance may present difficulties for kindergarten teachers evaluating FMS in kindergarten children in a classroom context [35].

Thus, the main objective of this study was to carry out a preliminary adaptation and validation of motor tests to assess the FMS associated with mathematical skills in the classroom context simultaneously for all students. For this purpose, firstly, it was necessary to identify the difficulties that kindergarten teachers have in objectively assessing the FMS of their students in the classroom context and to formulate criteria to select the motor tests that may present the least difficulties to educators for their application. Secondly, a description of the characteristics of the motor tests to assess FMS associated with mathematical skills, identified in the study by Flores et al. [14], was provided, and the tests that met a greater number of criteria were selected.

## 2. Materials and Methods

### 2.1. Sample

**Figure 1:**
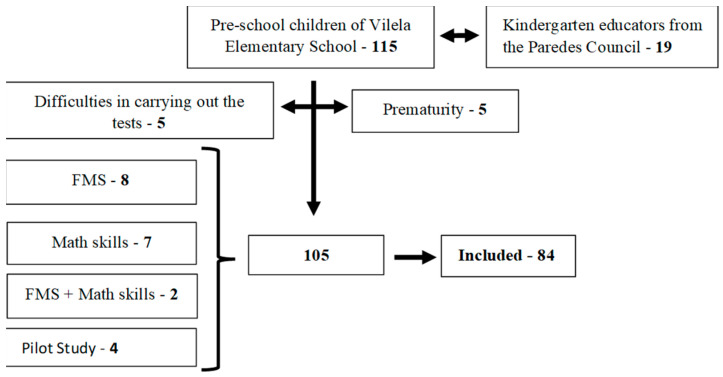
Sample flowchart.

In a first phase, with the aim of reporting the difficulties experienced by kindergarten educators assessing the FMS in their students in the classroom context, a sample of 19 kindergarten educators teaching in schools in the Municipality of Paredes (Porto, Portugal) was used, all female, aged between 44 and 60 years (mean age 50.2 years). For the preliminary validation of the degree of reliability and predictive criterion, between the motor tests adapted to assess the FMS and the assessment instrument of mathematical skills, 115 children attending preschool education at Escola Básica de Vilela, from the Agrupamento de Escolas de Vilela, Paredes (Porto, Portugal), were included. However, 5 children were excluded for not being able to perform the assessments and 5 for being born prematurely. Generally, among the most frequent problems in premature children are those associated with FMS and GMS [36,37], which negatively influence their academic performance [38,39]. Of the remaining 105 eligible children, 12 missed the FMS assessment, 7 missed the math skills assessment, and 2 missed both assessments. Therefore, the final sample consisted of 84 preschool children. Figure 1 shows the flowchart of the two study samples.

The 84 children were divided into 5 classes. The average age of the sample was 4.51±0.85 years. Regarding age distribution, the highest number was in the range of 5 years (40 children) and the lowest was 6 years (6 children) (Table 1).

The sample was significant of the population (*n* = 105) since for a confidence level of 95% and a margin of error of 5%, 83 children were required [*n*/(1 + *n* × e2) where *n* = 105; e = 0.05].

A request for authorization was sent to the parent so that the child could participate in the study. The request for authorization described all the tests that the child would perform and informed them that the data collected were confidential and only used in this research.

All procedures were in accordance with the Declaration of Helsinki for research in human beings.

### 2.2. Instruments

#### 2.2.1. Questionnaire to Kindergarten Teachers to Obtain Information on the Objective Evaluation of FMS in Their Students

In order to obtain information regarding the objective evaluations of FMS carried out by kindergarten teachers on their students, a questionnaire was prepared in which the first part intended to describe its purpose and the characterization and importance of FMS in mathematical performance. The second part consisted of four questions: question 1, “Do you objectively evaluate fine motor skills of your students in the classroom context?”. This question allowed us to identify how often kindergarten teachers evaluate the FMS of their students; question 2, “If you answered yes to the first question, what instrument do you use to carry out this evaluation?”. This question allowed us to identify the instruments used in this evaluation; question 3, “Have you ever used any of the instruments presented in the table to evaluate the fine motor skills of your students?”. This question allowed us to know if the kindergarten teacher had already administered any of the instruments identified in the study carried out by Flores et al. [14] to evaluate the FMS associated with mathematical skills; question 4, “What are the main difficulties you find in evaluating fine motor skills of your students in the classroom context?”. This question allowed us to identify the main difficulties that kindergarten teachers have when evaluating the FMS of their students in the classroom context.

At the end of the questionnaire, a space was reserved for kindergarten teachers to report the necessary observations.

#### 2.2.2. Evaluation of FMS Associated with Mathematical Skills

For the selection of motor tests to assess FMS, the tests identified in the study conducted by Flores et al. [14] were included.

To assess FMC, the following tests were included: Grooved Pegboard Test (GPT), which consists of fitting pins into holes [40]; inserting coins and threading beads from the manual dexterity scale, Band 1, of the Movement Evaluation Battery for Children, 2nd Edition (MABC-2) [41]; battery designed to provide an estimate of fine motor skills of preschool children (BEFMS), three tests, inserting pins, threading beads and turning blocks [42]; object manipulation subscale of the Learning Accomplishment Profile-Diagnostic, 3rd Edition (LAP-D) [43]; visuomotor accuracy subtest of the NEuroPSYchological evaluation battery, 2nd Edition (NEPSY) [44]; manipulation subscale of the Peabody developmental motor scale, 2nd Edition (PDMS-2) [45].

To assess VMI, the following tests were included: Fine Motor Scale, The Brigance Inventory of Early Development III—Standardized (IED III) [46]; Test of Visual–Motor Integration, 6th Edition (VMI) [47]; Copy Design Task (CDT) [48]; subtest design copying of the NEPSY [44]; writing subscale of the LAP-D [43]; visuomotor integration subtests of the PDMS-2 [45].

#### 2.2.3. Mathematical Skills Diagnosis

The Weschler Preschool and Primary Scale of Intelligence-Revised (WPPSI-R) [49] was used to diagnose mathematical skills. This scale was reviewed and adapted to the Portuguese population and covers the age groups from 3 to 6 years old and 6 months [50]. Applicators should be experienced and strictly follow the administration instructions and quotation. It can be applied anywhere, as long as it is quiet and free of external distractions (if possible, avoid placing the child in front of windows). It should be used at a table low enough so that the child can work comfortably and be seated on a chair, which allows the child to rest their feet on the floor. The applicator should sit in front of the child and provide a collaborative and cooperative environment so that the child feels comfortable and available to cooperate and respond to the demands imposed by the tests. The applicator should only start the test when the child is comfortable. During the application of the test, the applicator should demonstrate enthusiasm and interest in the child’s work, praising the child’s effort. However, they should not tell the child “That’s OK” or “That’s right” after a correct answer. If the child cannot answer a question, the applicator should encourage the child by saying “Try it” or “I bet you can do it, try again”. However, they should not insist excessively, as this could frustrate the child. Sometimes the child gives two or more answers to a question. In this case, if the child’s intention is to replace a previous answer, the initial answer is ignored and only the last one is scored. If the child simultaneously gives a correct and an incorrect answer, the applicator should ask them to choose one of the two.

The WPPSI-R is composed of two subscales, the Achievement subscale (comprising the subtests Composing Objects, Geometric Figures, Squares, Labyrinths, Completing Pictures and Animal Board) and the Verbal subscale (comprising the subtests Information, Comprehension, Arithmetic, Vocabulary, Similarities and Memorized Sentences). In this study, the Arithmetic test of Verbal subscale was used, where quantitative concepts, counting and problems, presented orally to the child, are evaluated. This test consists of 23 items divided into 3 parts. (i) Quantitative skills, picture items (items 1 to 7), where the child must point out, among a set of objects, visually presented, the one with a quantitative characteristic verbally stated. For this test, a large stimulus notebook with images is required. (ii) Numeracy skills, counting items (items 8 to 11), where the child should demonstrate numerical knowledge by counting and manipulating blocks. (iii) Problem-solving skills, verbal items (items 12 to 23), where the child should solve arithmetic problems presented orally.

The test should start at item 1. Although there is no time limit for the child to answer, after 15 s for items 1 to 11 and 30 s for items 12 to 23, a 0 score should be given and the applicator should move on to the next item. One point shall be awarded for each correct response (maximum score 23 points), and the test is interrupted after 5 failures. The raw scores are converted into standard scores, and a profile is obtained. Standardized results vary between 1 and 19, with 10 representing the mean value with a standard deviation of 3.

### 2.3. Procedures

The first objective (i) allowed us to obtain information from kindergarten teachers on the objective evaluation of FMS, in the classroom context, in their students. For this purpose, kindergarten teachers were informally asked to fill in the questionnaire (June 2022). We tried to have kindergarten teachers teach in different schools to check how many schools could evaluate FMS and avoid similar answers. The questionnaire was delivered in person, completed and collected on time. The results of the questionnaire allowed us to identify the main difficulties expressed by kindergarten teachers when objectively evaluating FMS of their students in the classroom context. These difficulties did not take into consideration the rigorous characteristics of the motor tests, only the kindergarten teachers‘ informed judgment. The difficulties expressed by the kindergarten teachers contributed to developing criteria with the aim of identifying the motor tests that presented fewer difficulties so that these can be applied simultaneously by kindergarten teachers to their students in the classroom context.

The second objective (ii) described the characteristics of the motor tests to evaluate FMS associated with mathematical skills identified in the study of Flores et al. [14] and identified and selected those that met a greater number of criteria.

**Figure 2:**
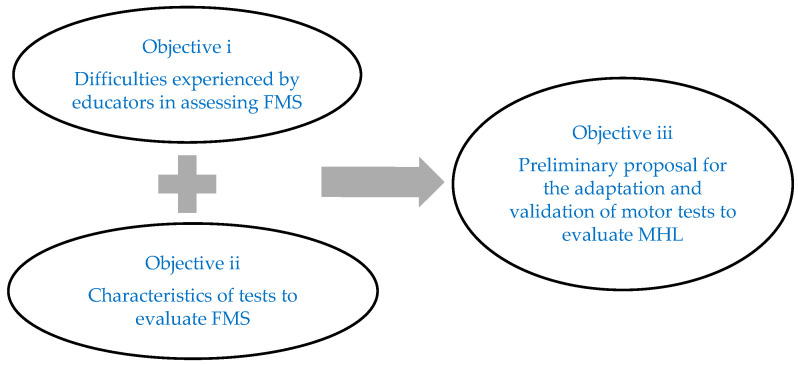
Summary of the study objectives.

After the selection of the tests, the third objective (iii) presented a preliminary proposal for the adaptation of the selected motor tests for the possibility of these tests being used by kindergarten teachers in the simultaneous evaluation of FMS in their students in the classroom context. After the adaptation, a preliminary validation of the tests was performed regarding their stability and predictive criterion validation. Figure 2 summarize the objectives.

The application of the adapted motor tests and the WPPSI-R was carried out by a single investigator (intra-observer). The investigator was the same person who carried out the preliminary adaptation of the motor tests in this study. To apply the WPPSI-R arithmetic test, he was instructed by a psychologist specialized in the area. The stability study aimed to measure the degree of reliability of the tests applied simultaneously at 2 different times to the class group (test-retest) and when applied individually. In the individual evaluations, for reasons of time, of the 84 children included in the study, 30 children (6 per class) were randomly selected in a systematic way (in an interval of 3 following the class list). The study of the predictive criterion validation between the adapted motor tests and the WPPSI-R arithmetic test aimed to analyze the association between FMS and mathematical skills.

Firstly, to test the adapted motor tests, a pilot study was carried out with 4 children, aged 3, 4, 5 and 6, different from those who made up the final sample. This study aimed to verify whether it would be necessary to make adjustments to the materials or procedures for the application of the adapted motor tests. Regardless of their age, no child showed any difficulty in understanding and performing the adapted motor tests; in this sense, there was no need to make any adjustments.

To measure the degree of reliability, and considering an interval of 10 to 14 days between the test-retest [51], the adapted motor tests were applied to the class simultaneously, during the morning period, on 16th March 2023 for the first time (test) and repeated on the 27th of March 2023 (retest). The simultaneous application of the adapted motor tests to the class, from the distribution of the materials to their collection, lasted a minimum of 20 min and a maximum of 27 min, with a longer period for the classes with younger children (3 and 4 years old). In turn, the individual application of the adapted tests was performed during the morning period on 30th March 2023. To measure the predictive criterion validity, the WPPSI-R Arithmetic test was applied on the 20th, 22nd, 23rd and 24th of March 2023.

### 2.4. Data Analysis

Descriptive statistics were used to describe the sample characteristics, namely frequencies, mean, standard deviation, minimum and maximum.

Prevalence, number (*n*) and percentage (%) were used to analyze the results of the questionnaire answered by the kindergarten teachers. In order to identify the motor tests, which were more adjusted to the objective of the study, we used the sum (∑) of the difficulties shown by the kindergarten teacher in the application of motor tests to evaluate the FMS of their students.

The intraclass correlation coefficient (ICC) was used to analyze the degree of reliability of the adapted motor tests [52]. Based on the 95% confidence interval of the ICC estimate, values less than 0.5, between 0.5 and 0.75, between 0.75 and 0.9 and greater than 0.90 are indicative of poor, moderate, good and excellent reliability, respectively [53].

To analyze the predictive criterion validity, Simple Linear Regression (Pearson—R2) was used, in which values equal to 2% were classified as a small effect, 13% as a medium effect and 26% as a large effect [54]. Since the studied variables obtained a normal distribution by the Kormorgonov–Smirvov test, Pearson’s correlation coefficient (r) was used to associate the variables. The magnitude of the correlation was classified as trivial (r ≤ 0.1), small (0.1 < r < 0.3), moderate (0.3 < r < 0.5), strong (0.5 < r < 0.7), very strong (0.7 < r < 0.9) or almost perfect (r ≥ 0.9) [55]. A 95% confidence level was set for a *p* < 0.05. Statistical analyses were performed using SPSS for Windows Version 26.0 (SPSS Inc., Chicago, IL, USA).

## 3. Results

### 3.1. Identification of Kindergarten Teachers’ Difficulties in Objectively Evaluating FMS of Their Students in the Classroom Context and Formulation of Criteria for Selection of Motor Tests

The first objective of this study was to verify whether kindergarten teachers objectively evaluated the FMS of their students in the classroom context, determine which instruments they used for that purpose and identify the main difficulties highlighted in the evaluation.

Regarding the first question, which aimed to identify the frequency of kindergarten teachers who evaluated FMS of their students in the classroom context, it was found that no kindergarten teacher did so (0%), thus they did not use any instrument for this purpose. This fact answered questions 2 and 3, which asked, respectively, which instrument they used to objectively evaluate FMS and whether they used any of the instruments identified in the study of Flores et al. [14].

Regarding question 4, it aimed to identify the main difficulties highlighted by kindergarten teachers when evaluating FMS of their students in the classroom context (Table 2).

The most frequent difficulties that justified not evaluating FMS in the classroom context were the lack of training (95%) and expensive material resources (95%). Also noteworthy is the fact that most of the instruments present many tasks to be evaluated (89%) and the lack of time (84%), which can be justified by the high number of students per class (74%). Another significant justification was the fact that the classes had students with very heterogeneous ages (63%). The lowest percentage was attributed to the lack of knowledge of the association between FMS and mathematical skills (47%) (Table 2). At the end of the questionnaire, there was a space for kindergarten teachers to describe any observations they considered relevant. Most kindergarten teachers (79%) explained that despite not evaluating FMS of their students, they do work a lot on these skills in the classroom, namely with tasks such as cutting with scissors, construction with different objects, buttoning and unbuttoning buttons, copying figures and shapes, drawing lines between spaces and between objects, painting, folding and collage and sticking objects in holes. Considering the results obtained by the questionnaire regarding the difficulties presented by kindergarten teachers in the evaluation of FMS, criteria were formulated to identify the motor tests with the most appropriate characteristics for kindergarten teachers to use in the evaluation of FMS simultaneously with their students in the classroom context. In this sense, the main difficulties highlighted were lack of training, lack of material resources, very complex instruments with many tasks to evaluate, lack of time, many students per class, very heterogeneous ages and lack of knowledge of the association between FMS and mathematical skills. Thus, for each of the difficulties highlighted by the kindergarten teachers, criteria were formulated to allow identification of the tests that would best fit the purpose of the study (Table 3).

Considering the kindergarten teachers’ difficulties, nine criteria were formulated, from A to I, to assist in the identification of the motor tests that best fit the purpose of this study (Table 3).

Thus, according to the characteristics of the tests, these were scored according to the formulated criteria (Table 3). If the criterion was respected, a point would be attributed, otherwise zero points. In this sense, the following score was proposed for each criterion:

Criterion A (number of tasks to apply)—tests that presented only one task to apply were scored one point, otherwise they were scored zero points;

Criterion B (number of criteria to score)—tests that presented more than one criterion to score were scored zero points;

Criterion C (scoring type)—only tests with a score based on quantitative evaluations were scored with one point;

Criterion D (materials for application)—tests that required cheap and easily accessible or acquired materials were scored one point;

Criterion E (application time)—only the tests that presented the shortest application time were scored with one point;

Criterion F (type of application)—only tests that allowed for simultaneous application to groups of students were scored one point;

Criterion G (age range)—only tests that allowed an application between the ages of 3 and 6 years old were scored with one point;

Criterion H (uniformity)—only tests in which the tasks to be applied would be the same regardless of age were scored with one point;

Criterion I (number of associated mathematical skills)—tests that were associated with a higher number of mathematical skills were scored with one point.

### 3.2. Characteristics of Motor Tests to Evaluate FMS in the Classroom Context and Identification and Selection of Those Who Obeyed a Greater Number of the Formulated Criteria

Another objective of this study was to describe the characteristics of the motor tests to evaluate the FMS and to identify and select those that complied with a higher number of criteria. For this purpose, initially, the characteristics of the tests to evaluate the FMC and the VMI were described and presented and, according to the criteria formulated, those which obtained a higher score were identified.

#### 3.2.1. Identification of Tests to Evaluate the FMC According to the Criteria Formulated

Table 4 presents the instruments and the description of the respective tests evaluating FMC used in association with mathematical skills [14].

Table 5 presents the specific characteristics of the tests that evaluated FMC according to the formulated criteria.

Thus, considering for this purpose the characteristics of the tests that evaluated the FMC, an attempt was made to identify those that met a greater number of formulated criteria (Table 6).

According to the data in Table 6, the highest scoring tests, and therefore those that can most easily be used to evaluate FMC, were the band 1: Manual Dexterity, insert coins and threading beads tests of the MABC-2 [41] and the visuomotor accuracy subtest of the NEPSY [44], both scoring 6 points.

#### 3.2.2. Identification of Tests to Evaluate VMI According to the Criteria Formulated

Table 7 presents the instruments and the description of the respective tests that evaluated VMI used in association with mathematical skills [14].

Table 8 presents the specific characteristics of the tests that evaluated VMI according to the criteria.

In this sense, considering for this purpose the characteristics of the tests that evaluated VMI, we tried to identify those that obeyed a larger number of the formulated criteria (Table 9).

According to the data in Table 9, the highest scoring tests, and thus those that can most easily be used to evaluate VMI, were the Visuomotor Integration Test (VMI) [47] and the Copy Design Task (CDT) [48], both with 6 points.

Once the tests were identified, it was necessary to select them.

The tests identified with characteristics offering a greater possibility of their use by early kindergarten teachers to evaluate FMC of their students were the band 1: Manual Dexterity insert coins and “threading beads” tests of MABC-2 [41] and the NEPSY visuomotor accuracy subtest [44]. With regard to the NEPSY visuomotor grip subtest, given its characteristics, it can easily be used by kindergarten teachers to evaluate the FMC, since only one task needs to be evaluated and it is the same for all ages, the evaluation is quantitative, it is indicated for children aged between 3 and 12 years, it only requires the use of pencil and paper and the application time is reduced (less than 5 min) [56]. However, a limitation of this test is that it requires three criteria to score: execution time, execution errors and the number of times the pencil is lifted. This limitation requires more experience and training by the kindergarten teachers, as well as more time to classify the results obtained. Another limitation is the fact that this test is very similar to those used to evaluate VMI, since it requires pencil and paper to perform it [14], whose objective is to quickly draw lines within paths/tracks that progress from wide to narrow and from straight to curves [44].

Regarding the band 1: Manual Dexterity, insert coins and threading beads tests of the MABC-2 [41], the characteristics are very similar to the visuomotor prehension NEPSY subtest [44], as they allow evaluating FMC in a short period of time (less than 5 min), the tests are applicable to children aged between 3 and 6 years and are the same regardless of age and the evaluation is quantitative. These tests, despite requiring more materials than the visuomotor accuracy NEPSY subtest, are easy to access and acquire. It should be noted that these tests only require one criterion to score (time in seconds), which facilitates the learning and training of kindergarten teachers to interpret the results. The characteristics of these tests are similar to others used to evaluate FMC since they require speed and manual dexterity based on observation of object-handling tasks with pincer-like movements [14].

In this sense, justifying the characteristics of the tests, the MABC-2 of band 1, threading beads and insert coins were the ones that best fitted the objective of the study and thus were the selected ones.

The insert coins test evaluates the ability to insert coins into a box as quickly as possible using first the dominant hand and then the non-dominant hand, scoring the fastest (6 coins for children aged 3 and 4 years and 12 coins for children aged 5 and 6 years). The threading beads test evaluates the ability to thread cubes on a string, with a metal pointed tip, as quickly as possible (6 cubes for children aged 3 and 4 years and 12 cubes for children aged 5 and 6 years). Studies have shown high correlations between the threading beads test and insert coins test [57,58,59]. High correlations between tests indicate that they are measuring the same abilities; on the other hand, if the correlations are low, they would be measuring different abilities [60]. In this sense, instead of using the two tests of band 1 of the MABC-2, it is justified to use only one to evaluate the FMC since it allows the reduction of material resources, application time, training for application and children’s learning to perform it. Thus, considering the test criteria regarding material resources, the threading beads test, rather than the insert coins test, is the one that best fits the objectives of this study. This option is justified by the fact that the materials for its application are easier to acquire; all that is needed is a string with a pointed end and cubes. In addition, although with different procedures and materials for application, this test is also used by other instruments to evaluate the FMC associated with mathematical skills, namely by BEFMS [42] and PDMS-2 [45]. For the application of the insert coins test, although the materials are also easy to acquire and the test only requires one box with a slit and coins to insert in it, taking into account the possibility of applying the test to a considerable group of children (a preschool class), it will be more difficult to acquire a large number of boxes with a slit and coins than to acquire cords and cubes. Regarding the time criterion, the insert coins test is applied twice, once on the dominant hand and once on the non-dominant hand, and the threading beads test is applied only once. In this sense, the threading beads test requires less application time.

The major limitation observed in the tests used to evaluate the FMC associated with mathematical skills was the fact that none of them allow the application to the class group simultaneously. This feature not only demands a lot of time from the kindergarten teacher to apply the tests to all students individually but also the impossibility to neglect the work to be done with the others who are not being evaluated. Even with this limitation, it does not mean that these evaluations should not be performed; however, the tests that best fit the reality and objective in question should be considered [61]. Thus, there is an urgent need to adapt a test to evaluate the FMC with the possibility of being easily applied to the whole class simultaneously, in a short period of time, with few material resources, where the results are objective (quantitative) and easy to interpret and classify. Regarding the tests identified with characteristics that offer a greater possibility of being more easily used by kindergarten teachers to evaluate VMI, they were the Visuomotor Integration Test (VMI) [47] and the Copy Design Task (CDT) [48]. These tests have very similar characteristics: they use paper and pencil to draw geometric shapes of increasing complexity; reduced application time (less than 5 min); only one criterion for scoring (execution error); quantitative evaluation (number of shapes correctly copied); it is the same test regardless of age; it covers all preschool ages.

One advantage of the CDT in relation to the VMI test is related to the number of geometric shapes to be copied, that is, the CDT only requires the copying of 8 figures and the VMI 15 figures. However, the VMI test is the only one that allows the application either individually or in groups of children [47]. This criterion is essential to substantially reduce application time, allowing the class to carry it out simultaneously and thus include all students in carrying out the same task. In this sense, this test may be the one that best fits not only the purpose of this study, but also the reality faced by kindergartens, since the VMI test does not require much training for its application, scoring and classification, it only requires paper and pencil to draw geometric shapes of increasing difficulty and can be administered to the whole class group in a short period of time [47].

Thus, considering the purpose of this study, the motor test identified as being more adjusted to preschool children’s reality to evaluate FMC is the threading beads test of band 1 of the MABC-2 [41] and to evaluate the VMI is the VMI test [47].

In this sense, there is an urgent need to adapt these tests in order to build an instrument to evaluate the FMS (FMC and VMI) associated with mathematical skills that has characteristics that easily allow an application by the kindergarten teacher simultaneously to their students, in a classroom context, in a short period of time, using few materials, with quantitative results and easy scoring and classification.

### 3.3. Adaptation and Preliminary Validation of the Motor Tests Selected to Evaluate the FMS Associated with Mathematical Skills

Another objective of this study was to present a preliminary proposal for the adaptation and validation of the selected motor tests to allow a simultaneous application by the kindergarten teacher to his/her students in a classroom context, in a short period of time and with few materials. The selected test best suited to the purpose of evaluating FMC was the MABC-2 threading beads test [41], and to evaluate VMI was the VMI test [47].

#### 3.3.1. Preliminary Proposal for Adapting the Threading Beads Test

The MABC-2 is one of the most used tests to identify children with developmental coordination disorder [62,63,64]. While some authors consider it a “gold standard” motor evaluation instrument [62,65], others emphasize the importance of finding more evidence before using it [64,65,66,67,68]. However, so far, there is no motor evaluation instrument referred by word-class criteria to diagnose children with motor disorder [69].

Considering the controversial literature on the adequacy of the MABC-2, the instruments should be suitable for the purpose of each study. Therefore, considering the purpose of this study, a preliminary proposal was made to adapt the threading beads test to allow not only an individual application, but, mainly, a simultaneous application to preschool students in the classroom by the kindergarten teacher.

Thus, based on the original threading beads test [41], adaptations were made to the materials to be used and the procedures for its application (Table 10).

The procedure regarding the arrangement of the tables in the classroom was added, as these should remain as per a normal school day so as not to influence or change the child’s routine in the classroom context.

Regarding the materials to be used, the blue mat from the original version was removed since this material is used to support the cubes and the string, thus, in this way, it can be replaced by the table itself where the task is performed. In addition, the conditions of the string were modified, as it does not necessarily have to be red with a metallic pointed tip, it can perfectly well be of another color and with a pointed tip of another material (e.g., plastic). A new scoring table was also proposed, considering the number of beads strung. These adaptations make it possible to remove the blue mat and create a more affordable acquisition of the cord, since this material is commonly used in everyday footwear. The proposed scoring table allows a quick reading and classification of the results.

Although the original test requires a short application time, it was only designed to evaluate children individually [41], thus the time spent on the application will depend on the number of children to be evaluated per class. Furthermore, when the kindergarten teacher applies the test individually, he/she only focuses on the child being evaluated, neglecting the remaining children. Therefore, it was considered an added value to present a preliminary proposal to adapt this test so that it could be applied simultaneously to the whole class group, not only reducing the application time, but also allowing all children to perform the same task at the same time (inclusive).

The most significant proposed adaptation to the original test was related to how to quantify the number of cubes strung on the string. The original test quantifies the time (in seconds) it takes for all of the cubes to be strung on the string. This procedure requires an individual evaluation, as the applicator should time it one at a time. Thus, to enable a simultaneous application to a group of children, it was necessary to adapt the test so that it could be performed within the same time interval by all children. Thus, the score will not be determined by the time it takes the child to string all the cubes, but by the number of cubes the child strings in a pre-set time interval. In this sense, the calculation to define the time to perform the test was obtained by taking into account the original cut-off points proposed by the authors for the maximum score in each age range (Table 11 and Table 12). Thus, the following times were proposed for carrying out the test: from 3:0 to 3:5 years old, 26 s; from 3:6 to 3:11 years old, 23 s; from 4:0 to 4:5 years old, 21 s, from 4:6 to 4:11 years old, 17 s; from 5 to 6:11 years old, 24 s. The application of the test should start with the oldest children (5 and 6 years old) and then follow the descending order of the age intervals (4:6–4:11; 4:0–4:5; 3:6–3:11; 3:0–3:5). This procedure, apart from saving on material resources for the test, allows the observation of the younger children, who consequently learn how to carry it out.

**Figure 3:**
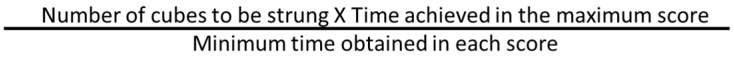
Formula used to convert original scores to proposals.

To obtain the scores in each age range, the total number of cubes to be strung was multiplied by the minimum time proposed for carrying out the test and the resulting value was divided by the shortest time that the child took to string the cubes to each of the cut-off points proposed by the authors [41]. The time was rounded up to the whole number obtained and equaled to the number of beads. Although, it was considered not to round down to the nearest whole number since the time would not be enough to fulfil the complete threading of the bead. Figure 3 shows the formula used to convert the original scores, taking into account the time in seconds it takes the children to string the cubes (6 or 12), to the number of cubes the child should string in the corresponding time interval.

For example, to apply the formula, a 5-year-old child who takes between 36 and 38 s to line up all 12 cubes will score 14 points. When applying the formula, to obtain this score, the child will need to line up 8 cubes (12 × 24/36 = 8).

Thus, the final score to be attributed will correspond to the number of cubes strung on the string in a stipulated time for each age. Table 11 and Table 12 show the conversion of the original scores of the threading beads test to the proposed adapted test taking into account the age of the child.

According to the MABC-2 authors [41], children at risk of motor disorder are those whose scores on the tasks evaluated are ≤7. Considering a score ≤7 as the cut-off point [41], which suggests that the child may have disorders in the FMC, then the score of new test suggests that children aged 3:0 to 4:11 years should string at least 2 cubes, with the exception of children aged 4:0 to 4:5 years, who should string 3 cubes, children aged 5:0 to 5:11 years, who should string at least 5 cubes and children aged 6:0 to 6:11 years, who should string at least 6 cubes.

Since the original score associated with the number of cubes strung in the adapted test for the child to be classified without motor disorder does not correspond to the same value for each age range, varying between 8 points (4:6 to 4:11 years) and 10 points (3:0 to 3:5 years; 4:0 to 4:5 years; 6 years), it was suggested to assign an equitable classification taking into account the original score on a scale from 0 to 4. Thus, the value 2 will correspond to the intermediate classification and suggests that the child is not at risk of showing a disorder in the FMC, values below 2 mean disorder (1 = moderate and 0 = severe) and values above 2 mean that the FMC is good or very good (3 = good; 4 = very good). In this sense, and according to the original scores, scores of 0 correspond to classification 0, between 2 and 7 corresponds to classification 1, between 8 and 10 to classification 2, between 11 and 13 to classification 3 and above 13 to classification 4 (Table 13). Table 14 presents the methods to apply the original VMI test [47] and the new proposed adapted test.

Having justified the advantages of the proposal in adapting the threading beads test, to be applied simultaneously to groups of preschool children, it was necessary to validate it and associate its result with mathematical performance.

#### 3.3.2. Preliminary Proposal for Adaptation of the Visuomotor Integration Test

The VMI test consists of the visuomotor integration test and two additional tests, the visual perception and the motor coordination tests [47].

The purpose of this study was to propose an adaptation only of the VMI test. According to the manual, the VMI test can be applied to small groups as well as individually. Generally, preschool children should be evaluated individually, however, a whole class may be evaluated if supervised by two adults [47].

As the aim of this study was to propose an adaptation of the VMI test for a simultaneous application to the class, the proposal was made only in terms of the materials and procedures of the test for application to groups. For this purpose, its short version was used, as this is the appropriate version for children attending preschool education, aged between 3 and 7 years [47]. Therefore, the aim of this test is to copy 15 geometric shapes as accurately as possible, in which the evaluation begins with copying a vertical line, a horizontal line and a circle. As the test progresses, the shapes become increasingly difficult to copy. The application of the test should follow the respective order of copying the shapes, otherwise the final result will be affected [47].

Considering the original VMI test regarding the materials and procedures for its application [47], this preliminary adaptation proposal aimed at reducing the amount of materials to be used and allowing a simultaneous application to the class group in a classroom context by only one adult (kindergarten teacher) (Table 14).

As for the threading beads test, the procedure related to the arrangement of the classroom tables was added, since they should be kept according to a normal day so as not to influence or change the routine of the children in the classroom context.

By observing Table 14, when analyzing the materials for application, classification and scoring of the test, these were kept according to the originals. However, it was considered necessary to create a table to convert the number of correctly copied shapes per age range to the “Average” performance profile. This table will allow the applicator (kindergarten teacher) to quickly classify the child’s performance profile as being above, equal to or below the “Average” performance profile.

When analyzing the materials for which the test has undergone changes, these are mainly justified by the change in the test booklets. The original proof given to the children to copy the shapes consists of seven sheets. The first sheet presents procedures for performing the test, the second sheet shows the 3 shapes for 2-year-old children to imitate and the following 5 sheets present the 15 shapes for the children to copy, 3 shapes per sheet [47]. This number of sheets when multiplied by the number of students per class and the number of times to administer the test, is probably a significant amount of resources (sheets). It was for this purpose, to save natural material resources, that this change is justified. Thus, it was proposed in the adapted test to use a single A4 sheet folded in half lengthwise to form four pages. The first one presents some procedures for taking the test, and pages 2, 3 and 4 present the 15 pictures to copy (5 pictures per page). To make this change possible, the squares in the original test of the shapes and the respective spaces to copy are 7.5 cm square, whereas in the adapted test the squares are only 5 cm square.

The VMI test can be applied and scored by any adult who is totally familiar with the materials and procedures and who has had practice supervised by an experienced applicator, since the test requires experience to interpret the results [47].

Younger children tend to develop very quickly, which is why the VMI test scoring norms are assigned at 2 month intervals. Shapes (carriers) can be used to compare with those copied by children, however, experienced classifiers rarely need these carriers to rate the shape held by the child [47].

The first attempt made by the child should always be graded. If the applicator does not realize which shape was made first, he/she should compare it with the size of the following shapes and try to identify the one that was made first. The scoring should end after three consecutive failed copied shapes. The classification and scoring criteria are those proposed by the authors (Table 15) [47].

The shapes to be copied are as follows (Table 16): 1—vertical line; 2—horizontal line; 3—circle; 4—horizontal cross; 5—oblique line to the right; 6—square; 7—oblique line to the left; 8—oblique cross; 9—triangle; 10—open square and circle; 11—three-line cross; 12—directional arrows; 13—rings in two dimensions; 14—six-circle triangle; 15—inclined circle and square.

Each correctly copied shape is awarded 1 point. The sum of the points corresponds to the natural score of the test. However, it is recommended that natural scores be converted into standard scores adjusted to each age interval [47]. In this sense, Table 16 shows the conversion of natural scores into standard scores for each age range from 3 to 6 years and 11 months, at intervals of 2 months. Based on the standard scores, a performance profile of the VMI test is assigned (Table 17).

An above average test score will indicate outstanding individual performance, compared to the normative population, and a below average score will indicate weakness [47]. For an easier and faster interpretation by the kindergarten teacher regarding the result obtained from the child about the number of shapes copied correctly, and taking into account the age range, the number of copies was associated with the average performance profile, taking into account the lower value of the cut-off points, which is 83 points (Table 18).

### 3.4. Preliminary Validation of the Motor Tests Proposed to Simultaneously Evaluate the FMS in the Classroom Context

After identification, selection and consequent preliminary adaptation of the motor tests, to evaluate the FMS, it was necessary to validate them. The degree of reliability of the tests was evaluated when applied at two moments simultaneously to the class group and individually, and a predictive criterion validation was also carried out to analyze the association between the adapted motor tests and mathematical abilities.

#### 3.4.1. Reliability Evaluation

To evaluate the degree of reliability of the adapted motor tests, the intra-observer test-retest method was used, including the intraclass correlation coefficient (ICC), with the objective of verifying its temporal stability. This method made it possible to analyze the consistency or homogeneity of the results of the same sample at two different points in time [70]. In order to measure the degree of reliability and considering that an interval of 10 to 14 days between test and retest [51] was adequate, the tests were applied to the class simultaneously, during the morning period, on the 16th of March 2023 (test) and repeated (retest) 11 days later. In turn, the individual application of the tests was performed during the morning period on the 29th of March 2023 (13 days after the test).

Table 19 presents the ICC results, by the test-retest method, as well as their lower and higher values and respective classification.

ICC values close to 1 mean that the measurements are very similar or homogeneous. For the classification of the ICC values, the Koo and Li [53] criteria were used, in which values less than 0.50 are considered bad, between 0.50 and 0.75 are moderate, between 0.75 and 0.90 are good and greater than 0.90 are indicative of excellent reliability. According to data in Table 19, the ICC values were all greater than 0.90, which means an excellent degree of reliability of the adapted threading teads test in relation to the test-retest, when the class group was evaluated simultaneously (ICC = 0.957) and individually (ICC = 0.924). Likewise, the result was similar for the adapted VMI test, as the degree of reliability was also excellent when the class group was evaluated simultaneously (ICC = 0.958) and individually (ICC = 0.961).

This result indicates that the tests adapted to evaluate the FMS, namely the threading beads test and the VMI test, showed an excellent degree of reliability and may therefore be administered simultaneously to the class group, since their results were stable between all the evaluations, group and individual.

#### 3.4.2. Predictive Criterion Validation

The predictive criterion validation aimed to analyze the relationship between the adapted motor tests, threading beads test and VMI test with the WPPSI-R arithmetic test. For this purpose, the scores and classifications obtained individually and the respective sum of the scores and classifications of the adapted motor tests were associated with the scores and classifications of the arithmetic test. The scores and classifications used in the association were the original ones for each test/proof, except for the classification of the threading beads test, which was adapted considering the original test score (Table 14). In this sense, the ratings assigned to all tests ranged from 0 to 4 (threading beads: 0 = severe; 1 = moderate; 2 = average; 3 = good; 4 = very good. VMI test: 0 = very low; 1 = low; 2 = medium; 3 = high; 4 = very high. WPPSI-R arithmetic test: 0 = very low; 1 = low; 2 = medium; 3 = high; 4 = very high).

Table 20 presents the results of the associations, from simple linear regression, between the scores and the classifications of the motor and mathematics tests, and Table 21 shows the respective standardized regression coefficients (β) and non-standardized regression coefficients (Beta).

By interpreting Table 20 and Table 21, a positive association (r > 0; Beta > 0) was found between the scores and respective classifications of the motor tests with the WPPSI-R arithmetic test. With the exception of the association between threading beads test scores and the arithmetic test, all other associations proved linearity by the F-test (*p* < 0.05), which means that all these associations were valid and the WPPSI-R arithmetic test result can be explained by the threading beads test score (F = 6.949; *p* = 0.010), the score and classification of the VMI test (F = 15.986; *p* < 0.001; F = 12.300; *p* = 0.001) and the sum of the scores and classifications of the threading beads test and VMI test (F = 17.012; *p* < 0.001; F = 16.657; *p* < 0.001). However, although the classification of the threading beads test was valid to explain the result of the arithmetic test (F = 6.949; *p* = 0.010; R^2^ = 7.8%), its correlation with this test was low (r = 0.280). These data suggest that the threading beads test, despite its classification, explains 7.8% of the result of the arithmetic test, and, according to Polit and Beck [54], this value is low. Thus, the threading beads test alone is not the most recommended test to explain the results of the WPPSI-R arithmetic test.

Regarding the adapted VMI test, the association of scores and classifications with the WPPSI-R arithmetic test, r = 0.404 and r = 0.361, respectively, in addition to proving the existence of linearity, explain 16.3% (R^2^ = 0.163) and 13.0% (R^2^ = 0.130) of the results of the arithmetic test. These values present an average effect [54], and the value of the correlations (r), according to Hopkins et al. [55], show a moderate effect. These results suggest that VMI test scores and classifications may be used to explain the results of the WPPSI-R arithmetic test.

Similarly, when adding the scores and classifications of the adapted motor tests, threading beads test and VMI test, the values of the association with the WPPSI-R arithmetic test increased (r = 0.415 and r = 0.411, respectively), as well as the influence on the arithmetic test (sum of scores: R^2^ = 0.172; 17.2%; sum of scores: R^2^ = 0.169; 16.9%).

The high variance in the difference between R^2^–R^2^(Aj) > 0.01 in all regression models is noteworthy, in which subtraction values lower than or equal to 0.004 (0.4%) would allow generalization. Thus, these data indicate that if these models were derived from the general population instead of a sample, they would not explain values lower than or equal to 0.4% of the variance, i.e., these models cannot be generalized to the general population.

## 4. Discussion

This study had as its starting point the survey of the difficulties found by kindergarten teachers when evaluating the FMS of their students in the classroom context. These difficulties made it possible to identify the motor tests that presented characteristics more suited to the needs of kindergarten teachers for this purpose. The lack of training together with the lack of material resources were the difficulties most reported by kindergarten teachers. Regarding material resources, this limitation could be overcome, as it would depend only on a monetary issue to solve the problem. However, with regard to training, most instruments to evaluate FMS have features that require significant expertise [34,35]. Thus, without such training, it is difficult for kindergarten teachers to have the knowledge of these instruments regarding their objectives, application procedures, scoring and classification [34]. A fact to highlight in the results obtained in the questionnaire to kindergarten teachers was the fact that, despite not carrying out FMS evaluations, they promote tasks in the classroom context with the aim of working and developing these skills. However, although it is considered very important to carry out these kinds of tasks in the classroom, it is hardly possible for kindergarten teachers to monitor the development of FMS in their students. On the other hand, preschool classes are made up of many students with very heterogeneous ages, which, given the characteristics of most instruments to evaluate FMS, practically makes their use by kindergarten teachers unfeasible, since, in addition to the necessary training, a lot of time will be required for this purpose [14]. Given the context, and the high number of instruments available for the same purpose [71], there was a need to select the motor tests that best fit the reality faced by kindergarten teachers on a daily basis to simultaneously evaluate FMS in the classroom context. Despite the difficulties, given their importance in the diagnosis of FMS, these evaluations should be performed [14] and the tests adjusted to their purpose [61].

The second objective of this study was to select and adapt the motor tests that showed characteristics that would allow an easier application by the kindergarten teachers simultaneously to the class in the classroom context. After demonstrating the results, to evaluate FMC, the threading beads test of band 1 of the MABC-2 Manual Dexterity [41] was selected, and the VMI test [47] was selected to evaluate VMI.

Regarding the threading beads test, despite the numerous advantages, the major limitation was related to the type of application since this test was designed to evaluate children individually [41]. This type of evaluation makes the kindergarten teacher focus only on the child being evaluated, neglecting the others, and it requires a long time to evaluate all the students. This limitation would make it practically impossible for the kindergarten teachers to evaluate the FMC. Since there is no world-class criteria-referenced motor test to diagnose children with motor disorder [69], and tests must be adapted to their purpose [61], there was a need to adapt the threading beads test. This adaptation allowed its simultaneous application to the class, reducing application time and allowing all the children to perform the same task at the same time. Thus, the most relevant adaptation proposal was related to how to quantify the number of cubes strung on the string, where the score to be assigned would not be determined by the time it would take the child to string all the cubes, but rather by the number of cubes the child would string on the string in a given time interval.

Motor evaluation studies using the MABC-2 have common features, i.e., they use the same procedures and standardized test scores, which emerged from the validation process of the instrument based on a sample in the UK [41]. In the MABC-2 manual, there is no evidence related to construct validity, and content validity was performed according to the evaluation of an evaluation committee based on the motor tasks of the first version of the MABC [72]. The implications of the abovementioned aspects demonstrate the need to restructure the instrument [73] with the withdrawal of some tests [74] and the reduction of the frequency of error in the final classification of the diagnosis of motor disorder in the children evaluated [75]. In a study carried out in China, the authors concluded that the reproducibility and validity of band 1 of the MABC-2 were poor, emphasizing the need to adjust part of the items to improve the psychometric properties of the test when applied to Chinese children aged 3 to 6 years [74]. For example, studies have shown that, in the Manual Dexterity dimension of band 1, the bike trail task showed low correlation with the threading beads task [57,74,76,77], and the reliability value increased when the bike trail item was removed from the analysis [63,64,68]. Regarding the classification of the child’s motor performance, a study carried out in the Netherlands [69], in which the original cut-off points for the diagnosis of motor disorder were maintained (≤7), the study concluded that there would be a higher number of children classified with motor disorder than expected, ranging from 16.2 to 31.3%, depending on the motor task evaluated. Thus, by maintaining the original standardization of the instrument, there is a possibility of underestimating children’s motor performance [69].

The adaptation of the threading beads test was justified given the divergence in the studies carried out with the MABC-2 regarding the procedures, scores and psychometric properties, which reported the need for a restructuring of the instrument [73]. In this sense, each test should be adjusted to each context, purpose, need and reality [61].

Regarding the adapted VMI test, its selection was mainly due to the fact that it can be applied either individually or in groups of children [47], which allows a significant reduction in the application time and ensures that all students perform the same task at the same time. The main adaptation was in terms of the materials to be used in the test since the original test consists of seven sheets and the adaptation allowed the use of only one sheet. This measure allows a very significant saving associated with natural resources.

Of the standard-referenced available tests, the VMI test [47] is one of the most widely used tools to evaluate VMI by occupational therapists [78,79,80], teachers and kindergarten teachers [81]. The VMI test is based on the combination of fine motor skills and visuospatial perceptual skills [82] and is composed of several subprocesses: perceiving and understanding spatial orientation, synthesizing parts into a whole, constructing and manipulating representations and reproducing models through controlled muscle movements [17,83]. The primary purpose of the VMI test is to help identify significant difficulties that some children have in VMI that may lead to learning, behavioral and neuropsychological problems. Through early identification, it is hoped that later difficulties can be prevented by appropriate educational, medical or other intervention [47]. Additionally, it can serve a wide variety of purposes in educational, neuropsychological and other forms of basic research [47].

The VMI test is designed to evaluate VMI on the premise that the whole may be greater than the sum of its parts, and the parts can work well independently, but not in combination. In other words, a child may have good visual and motor perception but show difficulties in integrating both parts [47].

The last objective of this study was to validate the tests through degree of reliability, by the test-retest method and predictive criterion validation, to analyze the association between adapted motor tests and mathematical abilities.

Regarding the degree of reliability, the results were excellent (ICC > 0.92), which means that the adapted motor tests, threading beads and VMI, can be administered simultaneously to the class group, since their results were stable between all the evaluations, group and individual.

Studies conducted with preschool children that examined the reliability of the age band 1 MABC-2 by the interobserver test-retest method obtained an intraclass correlation of good [59,84,85] to excellent [86]. A study carried out with children aged between 3 and 13 years obtained high intra- and interobserver reliability in all MABC-2 tests [87]. In addition, band 1 of the Spanish version of the MABC-2, which obtained adequate reliability, could be used to evaluate motor development in preschoolers [88]. In an investigation of the MABC-2, age band 1, according to its reliability (test-retest), in which 201 preschool children participated, the results indicated a quite acceptable ICC in the items observed in manual dexterity [89].

Regarding the VMI test, it was standardized and normalized 6 times between 1964 and 2010 with a US population of more than 12,500 children aged 2 to 18 years. The stability of the results by age group across reviews was certified and showed good psychometric properties, including a test-retest reliability of 0.88 and an interobserver reliability of 0.93 [47]. The validation of the VMI test was subsequently carried out in other European and South American countries, with a consensus on its validity and robustness as well as its resistance to cultural influences [90,91,92,93]. However, results from studies have shown different patterns in the VMI performance in different cultures, especially among preschool and school-aged children [94,95]. Thus, cultural variables can affect children’s performance in visuomotor skills evaluation tests [96]. However, the VMI test has provided evidence of its ability to identify academic problems [81,82], as VMI can influence mathematics performance in preschool children [27,28,29,30,31,32,33].

The predictive criterion validation aimed to analyze the relationship between the adapted motor tests, threading beads and VMI, and the arithmetic test of WPPSI-R. With the exception of the association between the threading beads test scores and the arithmetic test, all other associations proved linearity by the F-test (*p* < 0.05), which means that all these associations were valid, and the WPPSI-R score could be explained by the threading beads test score, VMI test score and classification and the sum of the threading beads and VMI test scores and classifications. Although the threading beads test score was valid to explain the result of the arithmetic test (F = 6.949; *p* = 0.010; R^2^ = 7.8%), its correlation with this test was low (r = 0.280). These data suggest that the threading beads test alone is not the most recommended test to explain the results of the WPPSI-R arithmetic test. On the contrary, VMI approves the association of scores and classifications with the WPPSI-R, in addition to proving the existence of linearity, they explained the results of the WPPSI-R by 16.3% and 13.0%, respectively, representing an average effect [54] and the value of correlations a moderate effect [55]. These results suggest that VMI test scores and classification can be used to explain the results of the WPPSI-R arithmetic test.

The literature has shown that VMI is, among the FMS, the one that most stands out in association with mathematical skills [14] and is also reported as an important factor for the diagnosis of mathematics learning difficulties [31]. Although our study also highlighted the adapted VMI test as the best predictor of mathematics performance, the threading beads test also contributed positively to association with mathematical skills. Some studies that included both FMS concluded that both FMC and VMI were predictors of mathematics performance [21,22,23,24].

According to Beery & Beery [82], VMI involves the integration of visual and motor skills coordinated through the fingers and hands, that is, the FMC. In this sense, FMC plays a very important role in school success [35,48,97], as children with better FMC may be better at manipulating objects, such as pencils or notebooks, which allows them to direct additional attention resources to learning rather than focusing them on movements associated with FMC [23]. In this sense, a child with a good FMC, when performing an academic task, may impose a lower cognitive load compared to a child who still shows difficulties in FMC [98,99].

However, the VMI is a complex and multifaceted construct that relies on both attention and FMC, as well as their integration, and as such is critical to adjustment to multiple aspects of school performance, including mathematics [17]. In a cross-sectional sample of 5- to 18-year-olds, Carlson et al. [17] found that VMI was associated with mathematics achievement, even after controlling for gender, socioeconomic status, FMC and intelligence quotient. The strong association between VMI and mathematics may arise because the components that are necessary for successful VMI are also implicated in mathematics learning [100,101,102]. In addition, neurobiological research indicates that the parietal cortex is an area of the brain that is particularly active during both VMI tasks and numerical processing [103]. Relatedly, VMI may contribute to the development of the mental number line [101] as well as to developing the understanding of part–whole relationships [33], both of which are important for mathematics performance. Research suggests that rudimentary FMC may not directly contribute to mathematics skills but rather may do so indirectly through other more complex skills [104], such as VMI. For instance, Sortor and Kulp [18] found that FMC was no longer significantly related to mathematics after controlling for attention and VMI in their sample of second through fourth graders. Similarly, FMC was not associated with mathematics achievement after controlling for VMI [17]. Although FMC is important for providing immediate access to mathematical learning through interacting with the environment [83], additional development beyond a certain skill level may not directly contribute to mathematics performance. Instead, FMC may be a prerequisite for other higher order cognitive processes, such as VMI and attention, which are more directly important for mathematics [105]. 

However, since the main objective of this study was to suggest a preliminary adaptation of motor tests that evaluated the FMS associated with mathematical skills to enable kindergarten teachers to apply them simultaneously to the class in a classroom context, the adapted VMI test seems to be the most adjusted to the reality faced by kindergarten teachers in their daily lives. Despite this suggestion, given the importance of the FMC in VMI [82] and the results evidenced in association with mathematical skills in preschool children [14], the kindergarten teacher may also include the adapted threading beads test to evaluate FMS.

## 5. Conclusions

This study aimed to perform a preliminary adaptation of motor tests to evaluate the FMS associated with mathematical skills to allow kindergarten teachers to apply them simultaneously to a preschool class, in a short period of time, with few material resources and easy access or acquisition and without the need for extensive training in test administration, scoring and classification. Although the threading beads test showed an excellent degree of reliability and a positive association with mathematical skills, the adapted VMI test, in addition to showing an excellent degree of reliability, showed a more robust result in the association with mathematical skills. In this sense, and according to the proposed objective, the adapted VMI test seems to be the most suitable one to be used by kindergarten teachers in the classroom to evaluate simultaneously the FMS associated with mathematical skills of their students.

## Figures and Tables

**Table 1:** Distribution of the number of students by age range and the respective average.

Age Range	*n*	Average	Standard Deviation
3 years old	14	3.07	0.02
4 years old	24	4.07	0.11
5 years old	40	5.05	0.03
6 years old	6	6.02	0.01
Total	84	4.51	0.85

**Table 2:** Difficulties shown by kindergarten teachers when evaluating FMS, in the classroom context, of their students.

Reasons for Non-Implementation	Number/Percentage
Lack of training.	18/95%
Expensive material resources.	18/95%
Instruments with many tasks to evaluate (complex).	17/89%
Lack of time.	16/84%
Too many students per class.	14/74%
Too heterogeneous ages of students to apply the same test.	12/63%
Lack of knowledge of the association between FMS and mathematical skills.	9/47%

**Table 3:** Formulation of criteria for the identification of motor tests that evaluated FMS associated with mathematical skills [14], taking into account the difficulties presented by kindergarten teachers in their application in the classroom context.

Difficulties of Kindergarten Teachers			Criteria for Identifying Motor Tests to Respond to the Difficulties Shown by Kindergarten Teachers
Lack of training	A	To apply (complexity)	Minimum number of tasks to apply. A minimum number of tasks generally requires less training for the applicator and less learning for the applicant.
	B	To score	The test must obey a minimum number of scoring criteria (example: execution time or number of executions or execution error, etc.). This criterion facilitates learning, analysis and interpretation of test results.
	C	Score type	It should be quantitative, and it allows a more objective evaluation and requires less experience since the final result will depend only on the performance of the evaluated and not on a subjective observation by the evaluator.
Material resources	D	Expensive	Inexpensive, easily acquired or accessed.
Many students	E	Lack of time	Evaluation should be as short as possible given the high number of students per class.
	F	Application type	Possibility of the test being applied to groups of students simultaneously. This criterion significantly reduces the application time.
Heterogeneous ages	G	Test age range	The tests should include the age range of 3 to 6 years. This criterion is justified by the fact that preschool education generally starts at age 3 and lasts until age 6 [13] before children start compulsory education (Schulman & Barnett. 2005).
	H	Uniformity	The test should be the same for all ages (3, 4, 5 and 6 years old), only the degree of difficulty should increase with increasing age. In this sense, children may perform the same task simultaneously in the classroom context—inclusive test.
Lack of knowledge	I	Association between FMS and mathematical skills	The test should be associated with a greater number of mathematical skills.

**Table 4:** Description of the tests used to evaluate FMC that were associated with mathematical skills in children of preschool education [14].

Instruments and Respective Tests	Tests Description
GPT [40]	It consists of a metal surface with a matrix of 5 by 5 keyhole-shaped holes in various orientations. During the task, the child is instructed to insert all the pins into the 25 holes, 1 at a time, as quickly as possible and from left to right, first with the dominant hand and then with the non-dominant hand.
MABC-2 Band 1—Manual Dexterity [41]	The insert coins and threading beads tests of band 1 (3–6 years) were used. Insert coins—insert coins into a box as quickly as possible. Children aged 3 to 4 years old, 6 coins; children aged 5 to 6 years old, 12 coins. They should use their dominant hand first and then their non-dominant hand, with the fastest being scored. Threading beads—thread cubes on a string, with a metal pointed tip, as fast as possible. Children aged 3 to 4 years old, 6 cubes; children aged 5 to 6 years old, 12 cubes.
BEFMS Tasks for Evaluating FMS [42].	Three tasks were used: pegboard, thread beads and turn the block. Pegboard task—insert up to 24 pins (4 cm long × 5 mm diameter), which are taken from a bowl, into a pegboard (wooden board) to form a line as quickly as possible. Before the child starts the task, the evaluator should demonstrate it by moving 3 pins, then the child should practice using 5 pins. The score represents the number of pins inserted in 35 s. String beads—children are instructed to string up to 20 beads (1.5 cm in diameter) on a metal rod (30 cm high), 1 at a time, which are inside a small container. If the child drops a bead on the floor, he/she must continue the task without collecting the dropped bead. Before the test, the evaluator should demonstrate the task with 3 beads, and the child should subsequently practice with 3 beads. Children’s scores represent the number of beads strung in 60 s. Turn the block—children must turn 16 small cylindrical blocks (4 cm in diameter and 3 cm high) that are inserted in slots in a wooden board. They must first turn the block to the opposite position and then turn it to the same position. Before the test, the evaluator should demonstrate the task with three cylinders and then the child practices also with three cylinders before starting the attempt. The final score is the number of cylinders turned over in 28 s.
LAP-D Manipulation Subscale [43]	It includes the tasks of building towers, steps and bridges with small tower blocks; threading string through holes; stringing beads on a string; turning pages of a book; placing pins on a pegboard; cutting with scissors; manipulating plasticine; and folding paper into different shapes. Evaluators are instructed to demonstrate the task first. Tasks are scored as correct (+) or incorrect (−). The final score is the total number of correct tasks.
NEPSY Visuomotor Accuracy Subtest [44]	Draw lines quickly within paths/tracks that progress from wide to narrow and from straight to curves. Scoring takes into account the time taken, total number of errors (number of times the line leaves the track) and total pencil lifts.
PDMS-2 Manipulation Subscale [45]	The child starts the test in the task adjusted to his/her age and continues in the sequence until failing the execution of three consecutive tasks. The motor tasks are picking up cubes, picking up the marker, buttoning and unbuttoning buttons and touching fingers. Initially, the ability to grasp an object with only one hand is tested, gradually evolving to finger involvement and bilateral activities. Each task is graded according to a fixed rating scale: 0 if the child cannot or does not attempt to perform the task; 1 if the child’s performance shows minimal proficiency or they do not complete the task; 2 if the child demonstrates optimal proficiency in performing the task.

Legend. BEFMS—battery designed to provide an estimate of children’s fine motor skills in preschool; LAP-D—Learning Accomplishment Profile-Diagnostic (Third Edition); GPT—Grooved Pegboard Test; MABC-2—Movement Evaluation Battery for Children (Second Edition); NEPSY—NEuroPSYchological evaluation battery; PDMS-2—Peabody developmental motor scale (Second Edition).

**Table 5:** Characteristics of the tests used to evaluate FMC that were associated with mathematical skills in children in preschool education [14] according to the formulated criteria.

Criteria									
Tests	A	B	C	D	E	F	G	H	I
GPT	1	1 (execution time)	Quantitative	Difficult access or acquisition	Less than 5 min	Individual	5 70	Equal to all ages	3
MABC-2 Band 1—Manual Dexterity	2	1 (execution time)	Quantitative	Easy access or acquisition	Less than 5 min	Individual	3 6	Equal to all ages	3
BEFMS Tasks for Evaluating FMS	3	1 (execution time)	Quantitative	Difficult access or acquisition	Less than 10 min	Individual	3 6	Equal to all ages	4
LAP-D Manipulation Subscale	28	1 (execution time)	Qualitative	Easy access or acquisition	More than 10 min	Individual	2.5 6	Age-adjusted	4
NEPSY Visuomotor Accuracy Subtest	1	3 (time, number and execution error)	Quantitative	Easy access or acquisition	Less than 5 min	Individual	3 12	Equal to all ages	3
PDMS-2 Manipulation Subscale	26	1 (execution error)	Qualitative	Easy access or acquisition	More than 10 min	Individual	0 5.9	Age-adjusted	2

Legend. A—number of tasks to apply; B—number of criteria to score the test; C—type of scoring; D—materials; E—application time; F—type of application; G—age range; H—uniformity of the test; I—number of associated mathematical skills; BEFMS—battery designed to provide an estimate of children’s fine motor skills in preschool; LAP-D—Learning Accomplishment Profile-Diagnostic (Third Edition); GPT—Grooved Pegboard Test; MABC-2—Movement Evaluation Battery for Children (Second Edition); NEPSY—NEuroPSYchological evaluation battery; PDMS-2—Peabody developmental motor scale (Second Edition).

**Table 6:** Scores of the tests used to evaluate the FMC that were associated with mathematical skills in preschool education children according to the formulated criteria.

Formulated Criteria	Instruments and Respective Tests					
	GPT	MABC-2	BEFMS	LAP-D	NEPSY	PDMS-2
	GPT	Band 1: Manual Dexterity	FMS Evaluation Tasks	Manipulation Subscale	Visuomotor Accuracy Subtest	Grip Subscale
A—number of tasks to be applied	1	0	0	0	1	0
B—number of criteria to score	1	1	1	1	0	1
C—type of scoring	1	1	1	0	1	0
D—materials	0	1	0	1	1	1
E—application time	1	1	0	0	1	0
F—type of application	0	0	0	0	0	0
G—age range	0	1	1	1	1	0
H—test uniformity	1	1	1	0	1	0
I—mathematical abilities	0	0	1	1	0	0
Score	5	6	5	4	6	2

Legend. BEFMS—battery designed to provide an estimate of children’s fine motor skills in preschool; LAP-D—Learning Accomplishment Profile-Diagnostic (Third Edition); GPT—Grooved Pegboard Test; MABC-2—Movement Evaluation Battery for Children (Second Edition); NEPSY—NEuroPSYchological evaluation battery; PDMS-2—Peabody developmental motor scale (Second Edition).

**Table 7:** Instruments and description of the respective tests used to evaluate the VMI that were associated with mathematical skills in preschool education children [14].

Instrument	Tests Description
IED III Fine Motor Subscale of the Physical Development Domain [46]	The tasks require the use of pencil and paper and consist of copying pictures, drawing a person, writing the sequence of numbers and sequential drawing of capital letters. The tasks to be completed depend on the age of the child. Children receive a score of 1 for each successfully completed task.
VMI Visuomotor Integration Test [47]	It requires the use of pencil and paper and requires the student to copy increasingly complex geometric figures. One point is awarded for each item correctly copied, and the test must be stopped after three consecutive failures. Children under 5 years old start the test at item 4, and those aged 5 or more start the test at item 7.
LAP-D Writing Subscale [43]	They include tasks that require the use of pencil and paper, such as copying numbers, letters and shapes and drawing simple objects such as people and houses. Items are scored as correct (+) or incorrect (−). The final score is the total number of correct items.
NEPSY Design Copying Subtest [44]	In this test, children use paper and pencil to copy two-dimensional geometric drawings of increasing complexity. The drawings are scored according to the established criteria, between 0 and 4 points for each of the 18 items (maximum score of 72). The test is stopped when the child incorrectly performs four consecutive items.
PDMS-2 Visuomotor Integration Subtests [45]	It consists of the tasks constructions with blocks (tower, train, bridge, wall, steps, pyramid); cutting with scissors imitating the horizontal line; threading beads; folding paper; copying (circle, cross, square); cutting paper (line, circle, square); lining with a string; putting small objects in a jar; drawing lines; connecting dots; coloring between the lines. Tasks are age-adjusted and placed in an increasing sequence of difficulty. The child starts the test on a specific item, according to his/her age, and continues in the sequence until he/she fails three consecutive ones. Each item is graded on a three-point evaluation scale: 0 = does not perform, 1 = minimum proficiency, 2 = optimal proficiency.
CDT Copy Design Task [48]	Instruct children to copy eight simple geometric designs. Children have two attempts at each drawing without applicator help. To each drawing is given a score of 1 if at least one attempt is correct, 2 if both attempts are correct, and 0 if both attempts are incorrect or not attempted. Item scores are summed and converted to a correct proportion of a possible score of 16.

Legend. CDT—Copy Design Task; IED III—the Brigance Inventory of Early Development III; LAP-D—Learning Accomplishment Profile-Diagnostic (Third Edition); NEPSY—NEuroPSYchological evaluation battery; PDMS-2—Peabody developmental motor scale (Second Edition); VMI—Test of Visual–Motor Integration.

**Table 8:** Characteristics of the tests used to evaluate FMC that were associated with mathematical skills in preschool education children [14] according to the formulated criteria.

Criteria Tests	A	B	C	D	E	F	G	H	I
IED III Fine Motor Subscale of the Physical Development Domain	9	1 (execution error)	Qualitative	Easy access or acquisition	More than 10 min	Individual	0 7	Age-adjusted	4
VMI Visuomotor Integration Test	15	1 (execution error)	Quantitative	Easy access or acquisition	Less than 5 min	Individual and groups	2 7	Equal to all ages	4
LAP-D Writing Subscale	28	1 (execution error)	Quantitative	Easy access or acquisition	More than 10 min	Individual	2.5 6	Age-adjusted	4
NEPSY Subtest Design Copying	18	4 (from execution error)	Quantitative	Easy access or acquisition	Less than 10 min	Individual	3 16	Equal to all ages	5
PDMS-2 Visuomotor Integration Subtests	72	3 (from execution error)	Quantitative	Easy access or acquisition	More than 10 min	Individual	0 5.9	Age-adjusted	2
CDT Copy Design Task	8	1 (execution error)	Quantitative	Easy access or acquisition	Less than 5 min	Individual	all	Equal to all ages	2

Legend. A—number of tasks to be applied; B—number of criteria to score the test; C—type of scoring; D—materials; E—time of application; F—type of application; G—age range; H—test uniformity; I—number of mathematical skills associated; CDT—Copy Design Task; IED III—the Brigance Inventory of Early Development III; LAP-D—Learning Accomplishment Profile-Diagnostic (Third Edition); NEPSY—NEuroPSYchological evaluation battery; PDMS-2—Peabody developmental motor scale (Second Edition); VMI—Test of Visual–Motor Integration.

**Table 9:** Scores of the tests used to evaluate the VMI that were associated with mathematical skills in preschool education children according to the formulated criteria.

Formulated Criteria	Instruments and Respective Tests					
	IED III	VMI	LAP-D	NEPSY	PDMS-2	CDT
	Fine Motor Subscale	Visuomotor Integration Test	Writing Subscale	Design Copying Subtest	Subtests of Visuomotor Integration	Copy Design Task
A—number of tasks to be applied	0	0	0	0	0	1
B—number of criteria to score	1	1	1	0	0	1
C—type of scoring	0	1	1	1	1	1
D—materials	1	1	1	1	1	1
E—application time	0	1	0	0	0	1
F—type of application	0	1	0	0	0	0
G—age range	1	1	1	1	0	1
H—test uniformity	0	1	0	0	0	1
I—mathematical abilities	0	0	0	1	0	0
Score	3	7	4	4	2	7

Legend. CDT—Copy Design Task; IED III—the Brigance Inventory of Early Development III; LAP-D—Learning Accomplishment Profile-Diagnostic (Third Edition); NEPSY—NEuroPSYchological evaluation battery; PDMS-2—Peabody developmental motor scale (Second Edition); VMI—Test of Visual–Motor Integration.

**Table 10:** Materials and procedures for the application of the original threading beads test [41] and preliminary adaptation.

Original Threading Beads Test	
Materials	Procedures
₋ Test instructions *; ₋ table and chair *; ₋ blue table mat ***; ₋ stopwatch *; ₋ yellow cubes with a hole in the center (3- and 4-year-old children use 6 cubes and 5- and 6-year-old children use 12 cubes) **; ₋ rounded red string with a pointed metal tip **; ₋ conversion table for test values **.	₋ Thread cubes onto the string as quickly as possible **; ₋ the task is evaluated individually **; ₋ the child should be sitting comfortably and with both hands resting on the proximal edges of the blue table mat, which is laid out on the table **; ₋ the cubes are horizontally aligned on the distal part of the blue mat and the cord is placed in the center of the mat **; ₋ the children are informed about the test procedures, and the applicator makes a demonstration with a bead and allows the children to replicate *; ₋ at the signal from the applicator, the child picks up a cube and starts to string it one at a time until the end of the string *; ₋ timing starts when the first hand leaves the mat and is stopped when the last cube passes through the metal end of the string **; ₋ the child has two attempts, and the best time in seconds is recorded **; ₋ if a mistake is made on the first attempt, the instructions and demonstration may be repeated, and a further attempt is allowed. If a second mistake occurs, it is noted as no score **.
Proposal for Adapting the Threading Beads Test	
Materials	Procedures
₋ Test instructions *; ₋ table and chair *; ₋ stopwatch *; ₋ wooden cubes with a hole in the center (3 and 4-year-old children use 6 cubes and 5- and 6-year-old children use 12 cubes) **; ₋ rounded string with a pointed plastic tip **; ₋ conversion table for test values **; ₋ score table +.	₋ The tables in the room should be arranged according to a normal school day +; ₋ start with 5- and 6-year-olds, followed by 4:6–4:11, 4:0–4:5, 3:6–3:11 and finally 3:0–3:5 +; ₋ thread as many cubes onto the string as possible in a pre-set time **; ₋ the task can be evaluated individually or in groups of children +; ₋ children should be seated comfortably and with both hands resting on the table **; ₋ the cubes are lined up horizontally in the center of the table and the string is placed in front and in the center of the cubes **; ₋ the children are informed about the test procedures, and the applicator demonstrates the test with a bead and allows the children to replicate *; ₋ at the applicator’s signal, the children take one cube and start to string one at a time until the end of the string *; ₋ timing starts when the first hand leaves the table and is stopped after the pre-set time **; ₋ the children have two attempts, and the highest number of beads inserted in the string in the two attempts is recorded **.

Legend. *—kept; **—modified; ***—removed; +—added.

**Table 11:** Conversion of the original scores of the threading beads test to the adapted test proposed for 3- and 4-year-old children.

Score	3:0 to 3:5		3:5 to 3:11		4:0 to 4:5		4:6 to 4:11	
	Original	Conversion	Original	Conversion	Original	Conversion	Original	Conversion
19								
18								
17								
16	<26	6 (6)						
15	27–32	5.77 (5)	<23	6 (6)	<21	6 (6)	<17	6 (6)
14	33–35	4.72 (4)	24–28	5.75 (5)	22–24	5.73 (5)	18–21	5.67 (5)
13	36–40	4.33 (4)	29–35	4.76 (4)	25–26	5.04 (5)	22–23	4.64 (4)
12	41–47	3.8 (3)	36–38	3.83 (3)	27–29	4.67 (4)	24–25	4.25 (4)
11	48–52	3.25 (3)	39–40	3.54 (3)	30–31	4.2 (4)	26–27	3.92 (3)
10	53–56	2.94 (2)	41–47	3.36 (3)	32–36	3.94 (3)	28–32	3.63 (3)
9	57–65	2.73 (2)	48–56	2.97 (2)	37–39	3.41 (3)	33–36	3.1 (3)
8	66–70	2.36 (2)	57–65	2.42 (2)	40–48	3.15 (3)	37–39	2.76 (2)
7	71–78	2.19 (2)	66–73	2.1 (2)	49–55	2.57 (2)	40–41	2.55 (2)
6	79–83	1.97 (1)	74–78	186 (1)	56–63	2.25 (2)	42–43	2.32 (2)
5	84–87	1.85 (1)	79–81	1.75 (1)	64–77	1.97 (1)	44–46	2.32 (2)
4	88–96	1.72 (1)	82–96	1.68 (1)	78–79	1.62 (1)	47–62	2.17 (2)
3					80–86	1.58 (1)	63	1.62 (1)
2								
1	+97	1.61 (1)	+97	1.42 (1)	+87	1.45 (1)	64+	1.59 (1)

Legend. Conversion—(number of cubes to be strung × minimum time taken to complete the test)/lower value of the time interval in each original score.

**Table 12:** Conversion of the original threading beads test scores to the proposed adapted test for 5- and 6-year-old children.

Score	5:0 to 5:11		6:0 to 6:11	
	Original	Conversion	Original	Conversion
19				
18				
17	<24	12 (12)	<24	12 (12)
16	25–29	11.51 (11)	25–28	11.52 (11)
15	30–35	9.6 (9)	29–31	9.93 (9)
14	36–38	8 (8)	32–33	9 (9)
13	39–40	7.38 (7)	34–35	8.47 (8)
12	41–43	7.02 (7)	36–37	8 (8)
11	44–47	6.54 (6)	38–42	7.57 (7)
10	48–49	6 (6)	43–45	6.69 (6)
9	50–53	5.75 (5)	46–47	6.26 (6)
8	54–55	5.33 (5)	48–49	6 (6)
7	56–60	5.14 (5)	50–54	5.76 (5)
6	61–66	4.72 (4)	55–58	5.23 (5)
5	61–66	4.72 (4)	59–63	4.88 (4)
4	67–96	4.29 (4)	64	4.5 (4)
3	97–121	2.97 (2)	65–73	4.43 (4)
2	122	2.36 (2)	74	3.89 (3)
1	122+	−de 2.36 (2)	74+	−de 3.89 (3)

Legend. Conversion—(number of cubes to be strung × time to perform the test)/lower value of the time interval of each original score.

**Table 13:** Proposal of the number of cubes strung, and respective score, for the adapted threading beads test, taking into account the execution time for the original cut-off points and proposal of a classification associated with the number of points obtained.

Ages			Number of Cubes Strung in the Adapted Test											
		0	1	2	3	4	5	6	7	8	9	10	11	12
Original score	3:0 to 3:5	0	6	10	12	14	15	16						
	3:6 to 3:11	0	6	9	12	13	14	15						
	4:0 to 4:5	0	5	7	10	12	14	15						
	4:6 to 4:11	0	3	8	11	13	14	15						
	5 years	0	0	3	3	5	9	11	13	14	15	15	16	17
	6 years	0	0	0	2	5	7	10	11	13	15	15	16	17
Proposed classification	3:0 to 3:5	0	1	2	3	4								
	3:6 to 3:11	0	1	2	3		4							
	4:0 to 4:5	0	1		2	3	4							
	4:6 to 4:11	0	1	2	3		4							
	5 years	0		1			2	3		4				
	6 years	0			1			2	3		4			

Legend. Rating—0 = severe; 1 = moderate; 2 = medium; 3 = good; 4 = very good.

**Table 14:** Materials and procedures for the application of the original VMI test [47] and proposed adapted test.

Original VMI Test—Administration to Groups	
Materials	Procedures
₋ Test instructions *; ₋ soft pencil (No.2) or pen *; ₋ table and chair *; ₋ test booklets **; ₋ natural scores and standard scores *.	₋ Distribute the test booklets and say: “Do not open the booklets until I ask you to”. “Place them with the hand facing upwards” **; ₋ check that children are comfortably seated and have the booklets centered. The applicator should demonstrate *; ₋ while doing the demonstration, he/she should say: “Open the booklet on page 3, page 2 has only blank squares like this”. “Page 3 has shapes in the top squares”. They should copy each shape in the space below. The proctor should demonstrate on the board how to copy the shapes, but never use a shape from the test **; ₋ tell them to copy the shapes in order, starting with number 4; ₋ tell them that some shapes are very easy, and some are very difficult even for an adult *; ₋ tell them that they have to copy all the shapes and cannot skip any of them *; ₋ tell them that they must do their best, both for the easy and the difficult shapes *; ₋ remind them that they only have one attempt at each shape and that they cannot erase them *; ₋ the test can end when the applicator feels that all the children have finished it. Usually, 10 min is sufficient. However, if any child has not finished, the examiner should allow the child to finish the test.
Adapted VMI Test Proposal—Administration to the Class	
Materials	Procedures
₋ Test instructions *; ₋ soft pencil (No.2) or pen *; ₋ table and chair *; ₋ examination paper **; ₋ natural scores and standard scores *. ₋ conversion table for the number of correctly copied shapes for the performance profile +.	₋ The tables in the room should be arranged as on a normal school day +; ₋ distribute the test sheet and say: “Do not open the sheet until I ask you to”. “Place it horizontally on the table with the arrows facing upwards” **; ₋ check that the children are comfortably seated with the paper centered. The applicator should demonstrate *; ₋ while doing the demonstration, say: “Open the sheet of paper, it has shapes in the squares at the top. You should copy each shape into the space below in order—from left to right and from top to bottom”. The applicator should demonstrate on the blackboard how to copy the shapes, but never use a shape from the test. They should make a complete replica of the test paper on the board to demonstrate **; ₋ they should tell them to copy the shapes in order, starting with number one, always keeping the arrows pointing upwards **: ₋ inform that there are also shapes on the back of the sheet to copy, and show the students +; ₋ you should inform them that some shapes are very easy and some are very difficult even for an adult *; ₋ inform them that they have to copy them all and not skip any *; ₋ inform them that they have to do their best, both for the easy and the difficult shapes *; ₋ remind them that they only have one attempt at each shape, and that they cannot erase it *; ₋ the test can end when the applicator feels that all the children have finished. Usually, 10 min is sufficient. However, if any child has not finished, the applicator should allow the child to finish the test *.

Legend. *—kept; **—modified; +—added.

**Table 15:** Summary of classification criteria in the execution of the 15 forms (adapted from Beery & Beery [47]).

N°	Shape	Criterion	With Score	No Score
1	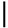	More than half of the line(s) within 30° of vertical.	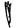	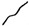
2	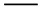	More than half of the line(s) within 30° of horizontal.	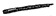	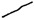
3	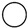	Any curve with a ratio of no more than 2 to 1 between its height and width.	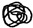	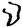
4	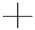	Two intersecting lines; all four “legs” at least 0.62 cm long (not including extensions); at least half of each line within 20° of the right angle.	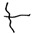	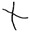
5	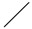	A “single” line (extensions are accepted); at least half of the line within 110°–160°; no abrupt change of direction.	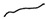	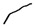
6	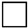	Four clearly defined sides (corners need not be angled).	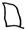	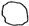
7	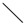	Four clearly defined sides (corners need not be angled). A “single” line (extensions are accepted); at least half of the line within 20°–70°; no abrupt change of direction.	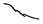	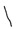
8	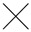	Two intersecting lines; angles formed by the lines between 20°–70° and 110°–160°; the longest of the four “legs” is not more than twice as long as the shortest (not including extensions).	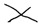	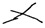
9	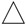	Three clearly defined sides; one corner higher than the others.	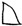	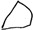
10	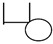	No more than 0.16 cm spacing or overlapping shapes; no big distortion in the open circle or square; height of circle and square within a 2 to 1 ratio; the bisector of the circle passing through the corner of the square should project into the square.	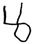	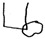
11	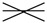	Three intersecting lines; the intersection gap is no more than 0.31 cm high; more than half of the horizontal line within 15° acceptable; more than half of both diagonals more than 10° from vertical.	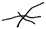	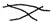
12	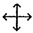	No tip inversion or “floating” tips; sharp points on arrows; no directional confusion; the length of the four “legs” is no more than two times the length of the shortest leg.	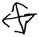	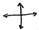
13	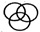	Three overlapping circles showing seven openings (the triangular opening in the center must be shown); one circle clearly below the others (the position must be checked by connecting the center points of the circles to form a triangle. The lower side of the triangle must be 20° or more above the horizontal).	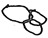	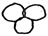
14	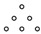	Six circles; baseline and at least one other correct side (a dashed line must touch at least the edge of each circle); baseline within 10° of horizontal; spacing between circles on the same side should be no more than 2 to 1.	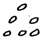	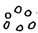
15	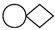	Square with four corners and a circle; opposite corners within 10° of vertical and horizontal; the square “touches” the circle with the closed corner; no more than 0.16 cm separation or overlap of shapes; corner contact in the middle third of the circle; height of circle and square in a ratio no greater than 2 to 1.	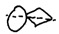	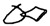

**Table 16:** Natural scores and standard scores (adapted from Beery & Beery [47]).

	Natural Scores (Number of Shapes Copied Correctly)														
Age	1	2	3	4	5	6	7	8	9	10	11	12	13	14	15
3-0|3-1	73	79	83	90	97	109	120	134	142	153	155				
3-2|3-3	71	76	81	88	94	106	116	129	137	147	155				
3-4|3-5	69	74	79	86	92	103	112	124	133	142	152	155			
3-6|3-7	67	72	77	84	90	100	109	120	128	137	146	155			
3-8|3-9	65	70	75	82	88	97	106	116	123	132	140	149	155		
3-10|3-11	63	68	73	80	86	94	102	111	119	127	135	142	150	155	
4-0|4-1	62	66	72	78	84	92	99	107	114	122	129	136	144	151	155
4-2|4-3	60	63	70	76	81	89	95	102	109	116	123	130	137	143	151
4-4|4-5	57	61	67	73	79	85	90	96	103	110	116	121	128	133	140
4-6|4-7	56	59	66	72	77	83	88	93	100	106	112	117	123	128	135
4-8|4-9	54	57	64	70	75	80	85	90	97	103	109	114	120	125	132
4-10|4-11	52	55	61	67	72	78	83	88	94	100	106	111	117	122	129
5-0|5-1	51	53	59	65	70	75	80	85	92	97	103	108	114	120	126
5-2|5-3	49	51	57	62	67	72	77	82	89	94	99	105	111	117	122
5-4|5-5	46	48	54	59	64	69	74	79	85	90	95	101	107	113	118
5-6|5-7	45	47	52	57	62	67	72	77	83	88	93	99	105	111	116
5-8|5-9		45	50	55	60	65	70	75	81	86	91	97	103	109	114
5-10|5-11		45	48	53	58	63	68	73	79	84	89	95	101	106	111
6-0|6-1		45	46	51	56	62	66	71	77	82	87	93	99	104	109
6-2|6-3			45	49	54	60	63	69	75	79	85	90	96	102	106
6-4|6-5			45	47	52	58	61	67	73	77	83	88	94	99	104
6-6|6-7				45	50	56	59	65	71	75	81	86	92	97	101
6-8|6-9				45	49	55	59	64	70	74	80	85	90	95	100
6-10|6-11				45	49	54	58	63	68	73	78	83	89	94	98

**Table 17:** Score and respective performance profile of the VMI test (adapted from Beery & Beery [47]).

Standard Score	Performance
133–160	Very high
118–132	High
83–117	Medium
68–82	Low
40–67	Very low

**Table 18:** Proposed conversion of the number of correctly copied shapes to the average performance profile as a function of the child’s age.

Age Range	3-0	3-2	3-8	4-2	4-8	5-0	5-2	5-8	6-0	6-8
	3-1	3-7	4-1	4-7	4-11	5-1	5-7	5-11	6-7	6-11
Number of shapes correctly copied	3	4	5	6	7	8	9	10	11	12

**Table 19:** Results of the intraclass correlation coefficient (ICC), by the test-retest method, obtained from the application of the adapted threading beads test and the adapted VMI test between the evaluation of the class group as well as individually.

Test-Retest	ICC	ICC Lower Limit	ICC Upper Limit	ICC Classification
Threading beads adapted: class group	0.957	0.934	0.972	Excellent
Threading beads adapted: individual	0.924	0.838	0.964	Excellent
VMI test adapted: class group	0.958	0.935	0.973	Excellent
VMI test adapted: individual	0.961	0.917	0.982	Excellent

Legend. ICC—intraclass correlation coefficient.

**Table 20:** Simple linear regression model between the scores and classifications obtained in the motor tests and the math test.

	*r*	R^2^	R^2^ (Aj)	R^2^–R^2^(Aj)	*F*	*p*
Threading beads score	0.165	0.025	0.015	0.010	2.291	0.134
Threading beads classification	0.280	0.078	0.067	0.011	6.949	0.010 *
IVM score	0.404	0.163	0.153	0.010	15.986	<0.001 **
IVM classification	0.361	0.130	0.120	0.010	12.300	0.001 **
Threading beads + IVM score	0.415	0.172	0.162	0.010	17.012	<0.001 **
Threading beads + IVM classification	0.411	0.169	0.159	0.010	16.657	<0.001 **

Legend. *—*p* < 0.05; **—*p* ≤ 0.001; F—F-ratio (significance of R^2^); *p*—significance level; r—Pearson’s correlation; R^2^—influence on WPPSI-R arithmetic test; R^2^–R^2^(Aj)—explains the generalization of the association.

**Table 21:** Coefficients of the simple regression model between the scores and classifications obtained in the tests and the mathematics test.

	β	Dp	Beta	*t*	*p*
Constant	8.129	0.992		8.192	<0.001 **
Threading beads score	0.170	0.112	0.165	1.514	0.134
Constant	1.219	0.239		5.098	<0.001 **
Threading beads score	0.304	0.115	0.280	2.636	0.010 *
Constant	1.692	1.989		0.851	0.397
IVM score	0.091	0.023	0.404	3.998	<0.001 **
Constant	1.126	0.213		5.298	<0.001 **
IVM classification	0.434	0.124	0.361	3.507	0.001 *
Constant	1.228	2.040		0.602	0.549
Threading beads + IVM score	0.088	0.021	0.415	4.125	<0.001 **
Constant	0.804	0.260		3.095	0.003 *
Threading beads + IVM classification	0.289	0.071	0.411	4.081	<0.001 **

Legend. β—non-standard coefficients; SD—standard deviation; Beta—standard coefficients; *t*—*t* test; *p*—significance level; *—*p* < 0.05; **—*p* < 0.001.

## Data Availability

Data are available upon request from the corresponding author.
